# More sun, less myopia: validating CUVAF as a biomarker for outdoor exposure in a cohort of Spanish schoolchildren

**DOI:** 10.3389/fmed.2026.1708836

**Published:** 2026-02-04

**Authors:** Miriam De La Puente, Cristina Irigoyen-Bañegil, Macarena Dosal-Franco, Sara Llorente-Gonzalez, Valentina Bilbao-Malavé, Martí Vinolas-Parcet, Jaione Bezunartea, María Hernández, Patricia Fernández-Robredo, Nerea Martín-Calvo, Alfredo García-Layana, Jorge González-Zamora, Jesús Barrio-Barrio, Sergio Recalde

**Affiliations:** 1 Department of Ophthalmology, Clínica Universidad de Navarra, Pamplona, Spain; 2 Retinal Pathologies and New Therapies Group, Experimental Ophthalmology Laboratory, Department of Ophthalmology, Universidad de Navarra, Pamplona, Spain; 3 Association of Magna Myopia with Retinopathies–AMIRES, Madrid, Spain; 4 Red Temática de Investigación Cooperativa en Salud: ‘Prevention, Early Detection, and Treatment of the Prevalent Degenerative and Chronic Ocular Pathology’ from (RD16/0008/0011), Ministerio de Ciencia, Innovación y Universidades, Instituto de Salud Carlos III, Madrid, Spain; 5 Department of Ophthalmology, Hospital Universitario Clínic, Barcelona, Spain; 6 Navarra Institute for Health Research, IdiSNA, Pamplona, Spain; 7 Faculty of Medicine, Universidad de Navarra, Pamplona, Spain; 8 School of Medicine, Department of Preventive Medicine and Public Health, Navarra Medical Research Institute (IdiSNA), University de Navarra, Pamplona, Spain; 9 CIBER Physiopathology of Obesity and Nutrition, Carlos III Health Institute, Madrid, Spain; 10 Department of Ophthalmology, Hospital Universitario de Navarra, Pamplona, Spain

**Keywords:** biomarker, children, CUVAF, myopia, outdoor activities

## Abstract

**Introduction:**

The increasing global prevalence of childhood myopia has become a significant public health concern. Among environmental risk factors, reduced outdoor activity (OA) has been consistently associated with a higher incidence of myopia. While questionnaires are traditionally used to estimate sunlight exposure, conjunctival ultraviolet autofluorescence (CUVAF) has emerged as an objective biomarker for cumulative ocular UV exposure.

**Methods:**

A cross-sectional case–control study was conducted among 2,616 children aged 7–8 and 11–12 years from 39 schools in Madrid, Spain. After reviewing the exclusion criteria, 354 children were removed from the study, leaving 2,262 participants in the sample. Participants were classified based on refractive error and underwent ocular examination and lifestyle questionnaires. The CUVAF area was measured in 1,129 children for logistical reasons, and they were grouped according to weekly time spent in OA (<7 vs. ≥7 h). Multivariate logistic regression and ROC curve analyses were performed to assess associations between OA, CUVAF, and myopia.

**Results:**

Myopia prevalence was significantly lower in children spending ≥7 h per week in OA than those with less exposure (9% vs. 18%; *p* < 0.001). CUVAF presence was strongly associated with higher OA and lower prevalence of myopia. A negative correlation was observed between CUVAF and myopia across schools (*r* = −0.6; *p* < 0.05). A diagnostic model using age-adjusted CUVAF showed a 91.9% probability of being non-myopic (negative predictive value) (positive predictive value: 10.79%; sensitivity: 76.5%; specificity: 29.6%). Each additional hour of OA reduced myopia risk by 2% (OR = 0.98; *p* = 0.01), while ≥7 h weekly halved the risk (OR = 0.50; *p* < 0.001). Conversely, the prevalence of high CUVAF increased with greater OA (OR = 2.54 for ≥7 h/week).

**Conclusion:**

CUVAF is a reliable, objective, and non-invasive biomarker of outdoor light exposure that correlates inversely with myopia in children. The larger sample size of this study supports the potential clinical utility of CUVAF not only for evaluating an individual’s risk of myopia development during childhood but also for monitoring adherence to lifestyle modifications aimed at its prevention. Its integration into routine screening could improve early detection and personalized prevention strategies for childhood myopia.

## 1 Introduction

Myopia is the most prevalent refractive error (RE) worldwide, causing a significant public health burden (1, 2). Over the last six decades, the number of individuals with myopia in Europe has tripled, while it has increased five-fold in Asia (3). By 2050, it is estimated that approximately 50% of the world’s population will have myopia (4). In addition, approximately 10% of patients will have high myopia (HM) based on the World Health Organization (WHO) estimations (4–9). Among children, the highest prevalence of myopia is in East Asia and Singapore (60%), while the prevalence of myopia in Europe is lower (40%) (2, 10).

Early-onset myopia progresses into adulthood and increases the risk of HM, which is a risk factor for glaucoma, cataracts, retinal detachment, and myopic maculopathy (11–13). Consequently, the WHO has identified myopia prevention as a priority in global efforts to prevent blindness (14), owing to its threat to ocular health in later life (15).

The etiology of myopia is multifactorial, encompassing genetic and environmental influences. Among the modifiable factors, time spent on outdoor activities (OAs) is protective, while near-vision activities (NVAs) are a significant risk factor (13, 16–25).

Epidemiological studies commonly rely on questionnaires to assess the time spent outdoors (8, 9, 26, 27); however, this approach is susceptible to recall bias and inaccuracies (8, 17, 28). To address this limitation, research has explored the use of potential biomarkers of outdoor exposure, such as conjunctival ultraviolet autofluorescence (CUVAF) area measurement (29). The CUVAF area refers to a localized area of autofluorescence in the bulbar conjunctiva observed under UV light that is attributed to sunlight exposure (30–35).

There is an inverse correlation between an individual’s RE and the CUVAF area (8, 36), which was confirmed by a meta-analysis on the association between the CUVAF area and myopia across different geographical regions and age groups (29). While that research focused on adults and young adults, investigations in pediatric populations reported an inverse relationship between CUVAF and myopia severity, suggesting that CUVAF may be an objective biomarker for managing childhood myopia. Notably, a predictive formula based on age was developed to estimate the expected CUVAF area, with values above a specific threshold considered protective against myopia progression (37). Therefore, this study aimed to validate the utility of CUVAF and the novel predictive formula in a larger pediatric cohort.

## 2 Materials and methods

### 2.1 Study design and ethics approval

A case–control observational study was conducted among children aged 7–8 and 11–12 years (38). Data collection was performed between September 2021 and March 2022. To ensure socioeconomic diversity, participants were recruited from 39 schools, including public, private, and subsidized schools, in the community of Madrid (Spain). Stratification by district was performed based on the segmentation previously applied in several studies conducted in Madrid Salud (38). All evaluations were conducted on-site by healthcare professionals.

This study was approved by the Ethics Committee (No. CUN.2019.155) of the Clínica Universidad de Navarra, in accordance with the principles of the Declaration of Helsinki. All participants were fully aware of its purpose and procedures, and written informed consent was obtained from their legal guardians.

### 2.2 Inclusion and exclusion criteria

The inclusion criteria were children aged 7–8 years and 11–12 years. The exclusion criteria were children with astigmatism or anisometropia ≥2.0 diopters (D), amblyopia, congenital myopia or myopia associated with another pathology, other ophthalmic diseases that may affect visual acuity or interfere with the performance of tests, and those with any conjunctival alteration or previous conjunctival surgery, among others that may lead to difficulty measuring the CUVAF area.

### 2.3 Data collection

Initially, 2,616 children were identified for inclusion in the study. To measure the RE, all participants underwent an initial evaluation with a photorefractometer (Plusoptix, Plusoptix GmbH, Nuremberg, Germany) at a distance of 1 m. Children with a RE of ≤0 D underwent cycloplegic testing (45 min after instillation of three drops of a cycloplegic with a 10-min interval) to avoid errors due to accommodation. This was followed by an ophthalmological examination by ophthalmoscopy and measurement of refraction with an autorefractometer (Myopia Master, OCULOS Iberia S. L., Tres Cantos, Madrid). Members of the research team, including two experienced ophthalmologists, two qualified optometrists, and three laboratory technicians, were trained before the study started. All instruments were checked and adjusted before examinations.

The spherical equivalent (SE) was calculated by summing the cycloplegic spherical refraction and half the cycloplegic cylindrical refraction. Given the high correlation between the right and left eyes, the mean of both eyes was taken as the reference value for the analysis. Participants were classified into control (SE > −0.50 D) and myopic groups (SE ≤ −0.75 D). Patients with myopia were further classified as “Myopic 1” (M1; −0.75 to −3.00 D), “Myopic 2” (M2; −3.25 to −5.75 D), or “High Myopic” (HM; ≤ − 6 D or ≥26 mm of axial length; Table 1).

**Table 1:** Cohort classification based on the spherical equivalent (SE) and the axial length (AL).

Classification criteria	Controls	M1	M2	HM
Spherical equivalent (SE)	> − 0.50D	−0.75 to −3.00 D	−3.25 to −5.75 D	≤ − 6 D or ≥26 mm of AL

Days before the ophthalmologic examination (between 7 and 10 days), participants or their guardians completed a questionnaire that included questions about family ocular history (myopia and HM in parents), personal history (previous refractive correction, amblyopia, use of glasses, or contact lenses), and lifestyle (time spent on OAs or NVAs as well as sun exposure habits).

**Figure 1:**
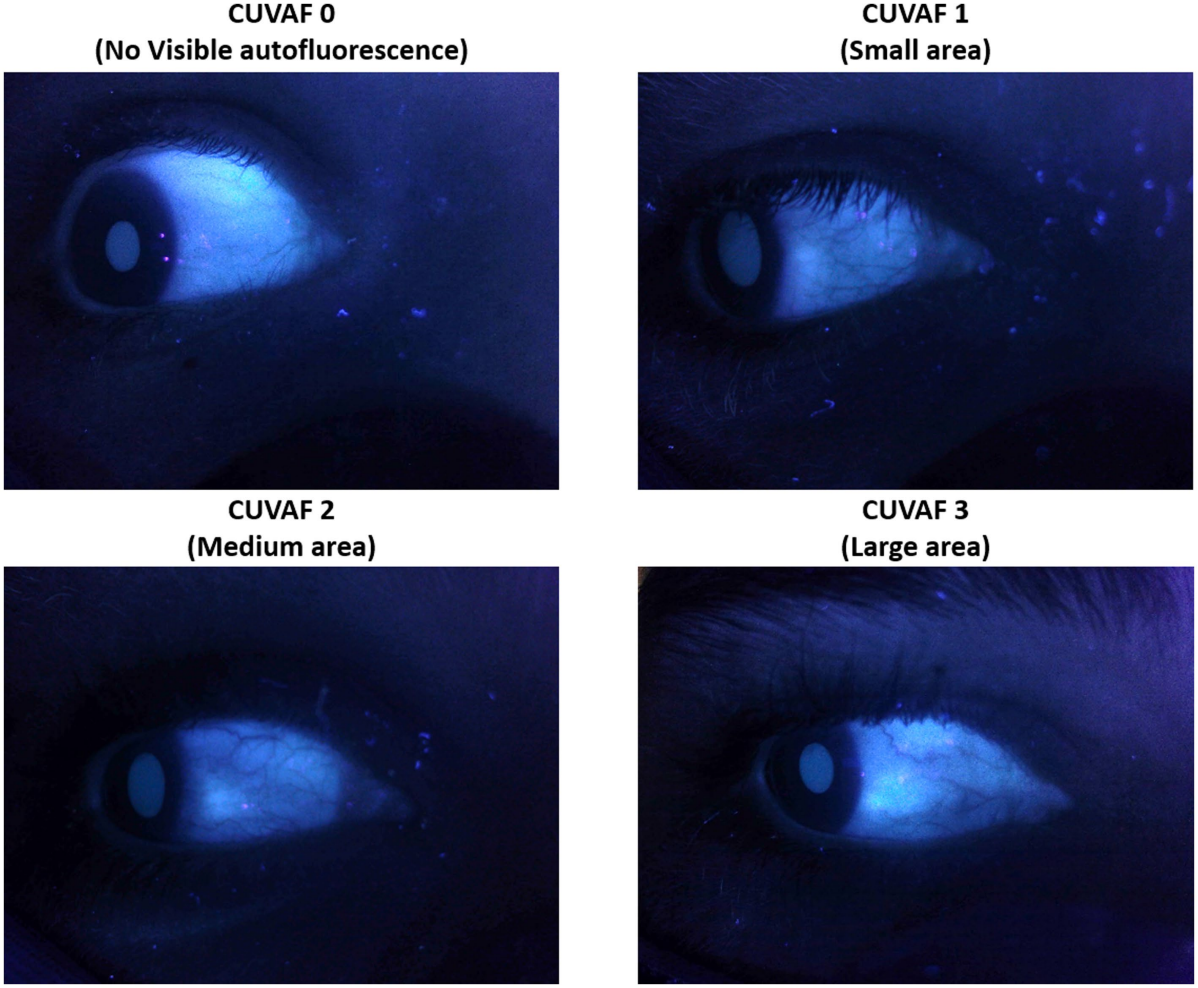
Scale used to classify the participants based on the CUVAF area.

The CUVAF area was assessed in the nasal and temporal zones of the bulbar conjunctiva of each eye using a UV light lamp. Two specialists independently categorized the CUVAF into three groups based on visual evaluations of the autofluorescence area at that time. Subjects were assigned to group 1, 2, or 3 according to whether they exhibited a smaller, intermediate, or larger CUVAF area, respectively. In case of disagreement, an independent third specialist decided on the category to which the subject belonged. The classification was guided by reference images (Figure 1). After classifying the participants into the three corresponding groups, a specific CUVAF area was assigned to each predefined group, and this assigned value was used for the subsequent analyses (1, 2, or 4 mm, respectively).

### 2.4 Statistical analysis

All information was stored in accordance with data protection laws and grouped into variables for subsequent analysis. After assessing the normal distribution of the sample using the Shapiro–Wilk test, the general characteristics of the participants were compared using the Mann–Whitney U-test for continuous variables, and the chi-square test was used for categorical variables since the expected values for each cell exceeded five due to the large sample size. Pearson’s correlation tests were performed between the CUVAF area and SE.

A previously published diagnostic formula was validated using this cohort. The predicted CUVAF values were calculated and compared with the observed outcomes, and sensitivity and specificity were determined at the optimal cutoff (95% confidence interval) based on the age-adjusted CUVAF area.

For all statistical analyses, the *α*-error was established at a *p*-value of < 0.05 (two-tailed). All statistical analyses were performed using STATA (version 19; StataCorp LLC, College Station, Texas, United States) and GraphPad Prism Software version 5.0 (GraphPad Software Inc., San Diego, CA, United States).

## 3 Results

### 3.1 Demographic characteristics

Overall, 2,616 children were recruited, including 1,234 and 1,382 children in second (7–8 years old) and sixth (11–12 years old) grades, respectively. Children with missing data on any key variable or covariable were excluded from the final analysis. After data cleaning, 2,262 participants were divided into two groups: 360 and 1,902 children spent <7 and >7 h/week in OAs, respectively. Significant differences were observed in age (*p* < 0.001), sex (*p* < 0.001), family history of myopia (*p* < 0.001), and district (*p* < 0.001) between groups. The demographic characteristics of the participants are shown in Table 2. Supplementary Table 1 shows the variable distribution before data cleaning.

**Table 2:** Demographic characteristics of the participants.

Characteristic	Time spent on outdoor activities		*p*-value
	<7 h/week	≥7 h/week	
N	360	1902	
Age, years	9.93 (2.04)	9.22 (2.10)	<0.001
Sex, male	135 (37.5%)	1,019 (53.6%)	<0.001
Family history of myopia, yes	118 (32.8%)	996 (52.4%)	<0.001
Type of School			<0.001
Public	159 (44.2%)	636 (33.4%)	
State-subsidized private	165 (45.8%)	942 (49.5%)	
District			<0.001
District 1	19 (5.3%)	176 (9.3%)	
District 2	90 (25.0%)	779 (41.0%)	
District 3	150 (41.7%)	665 (35.0%)	
District 4	101 (28.1%)	282 (14.8%)	
Screen exposure, *hours/week*	19.6 (14.9)	21.2 (15.5)	0.075
Spherical equivalent	−0.41 (1.05)	−0.17 (0.76)	<0.001

Comparisons are between <7 and ≥7 h spent on outdoor activities.

### 3.2 Factors associated with differences in myopia prevalence

**Figure 2:**
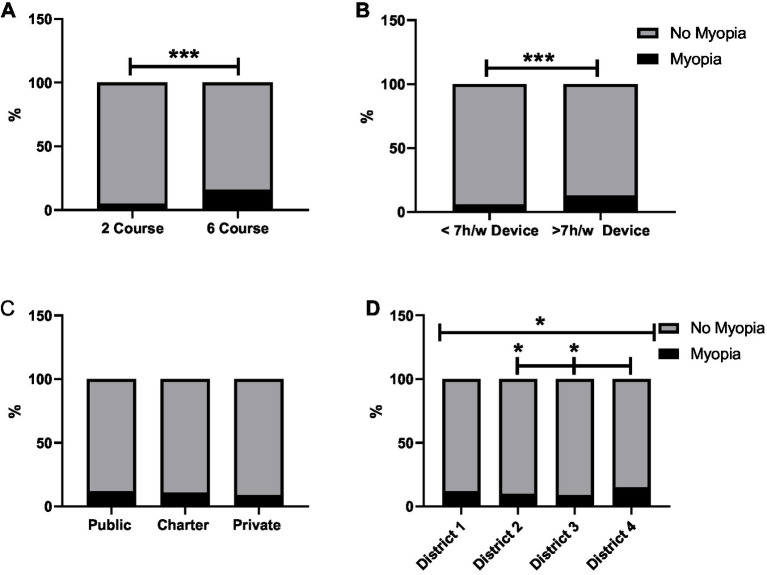
Relationship between myopia and different variables. **(A)** Percentage of children among second and sixth graders. **(B)** Difference in myopia prevalence according to the number of hours spent with electronic devices. **(C)** Difference in myopia prevalence between different school types. **(D)** Difference in myopia prevalence in different districts. *p < 0.05, **p < 0.01, and ***p < 0001.

Children aged 7–8 years showed a lower prevalence of myopia compared to those aged 11–12 years (5% vs. 16%; *p* < 0.001; Figure 2A). Similarly, children who spent >7 h/week using digital devices had a significantly higher prevalence of myopia than those with less screen time (13% vs. 6%, *p* > 0.001; Figure 2B).

No significant differences in myopia prevalence were observed between public (12%), charter (11%), and private (9%) schools (*p* > 0.05; Figure 2C). In contrast, there were significant differences in myopia prevalence between districts, with the highest rates in district 4 (15%), followed by districts 1 (12%) and 2 (10%), and the lowest in district 3 (9%) (*p* < 0.05; Figure 2D).

### 3.3 Relation of OAs, CUVAF, and myopia

**Figure 3:**
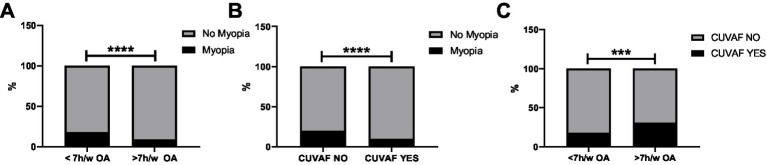
Analysis of the presence or absence of myopia/CUVAF in different groups. **(A)** Comparison of the presence or absence of myopia according to the amount of exposure to outdoor activities. **(B)** Comparison of the presence or absence of myopia according to the presence or absence of CUVAF. **(C)** Analysis of the presence or absence of CUVAF according to the amount of exposure to outdoor activities. ***p < 0.001 and ****p < 0.0001.

Of the total participants, 1,129 children underwent CUVAF measurements at schools where such measurements were permitted. Children who spent >7 h/week on OAs had a significantly lower prevalence of myopia compared to those with less OAs (9% vs. 18%; *p* < 0.001; Figure 3A).

Myopia was significantly associated with CUVAF values, with children in the myopic group showing a lower CUVAF percentage than those in the non-myopic group (10% vs. 20%; *p* < 0.001), indicating a relationship between reduced light exposure and myopia (Figure 3B).

Furthermore, this association was also consistent with time spent on OAs, as higher CUVAF values were noted in children who spent >7 h/week on OAs compared to those with less exposure (31% vs. 18%; *p* < 0.01; Figure 3C). This finding reinforces the association between reduced outdoor exposure and increased risk of myopia through self-reported OAs and objective biomarkers.

### 3.4 Factors associated with differences in CUVAF prevalence

**Figure 4:**
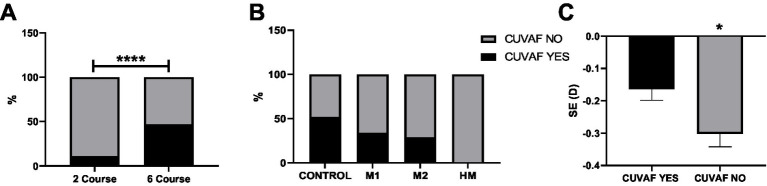
**(A)** Comparison of the presence of CUVAF between second and sixth graders. **(B)** Comparison of the presence of CUVAF between the control and myopic groups (controls vs. M1, M2, and HM). **(C)** Comparison of the SE between the CUVAF (+) and (−) groups. *p < 0.05 and ****p < 0.0001.

There was a significant association between grade and CUVAF prevalence. Children aged 7–8 years (second grade) had significantly less CUVAF than those aged 11-12 years (sixth grade) (11% vs. 47%, *p* < 0.0001; Figure 4A). Moreover, the presence of CUVAF displayed a tendency across myopia grades, with greater prevalence in non-myopic individuals; however, the difference was not significant (C: 52%, M1: 34%, M2: 29%, and HM: 0%; *p* > 0.05; Figure 4B). This finding is similar to the differences in CUVAF and SE, with the CUVAF (+) and (−) groups showing a mean SE of −0.17D and −0.3D, respectively (Figure 4C).

**Figure 5:**
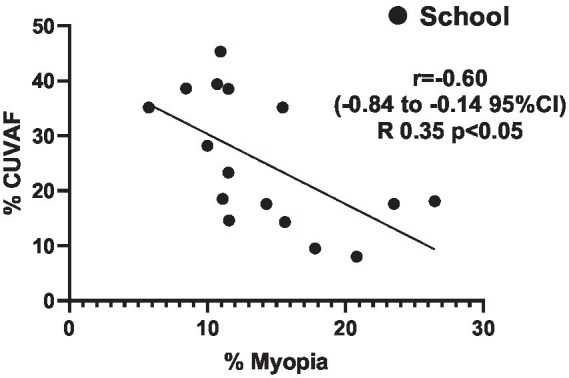
Correlation analysis shows an inverse correlation between the percentage of 12-year-old children with CUVAF and myopia prevalence across schools (*r* = −0.6, *R*^2^ = 0.35, *p* < 0.05).

Additionally, there was a moderate-to-strong inverse correlation between the percentage of 12-year-old children with CUVAF and the prevalence of myopia across schools (*r* = −0.6; *p* < 0.05; Figure 5), suggesting that a longer time spent on OAs, as indicated by CUVAF, may be associated with lower rates of myopia.

We then developed a model to assess the diagnostic performance of CUVAF in identifying children without myopia using standard classification metrics. The model was adjusted for age, sex, district, type of school (public vs. state-subsidized private), family history of myopia (yes vs. no), and electronic device use (<7 h/week vs. >7 h/week). The test showed good sensitivity (76.5%) and low specificity (29.6%), indicating a reasonable ability to identify children with myopia, although with limited precision in excluding false positives. The negative predictive value was very high (91.9%), indicating that a majority of children identified as CUVAF (+) were correctly classified as non-myopic and supporting the potential utility of CUVAF as a non-invasive screening tool for myopia risk in school-aged populations. Conversely, the positive predictive value was 10.79%, indicating that only 10.79% of children identified as being CUVAF (−) were actually myopic (Table 3). In this regard, the association between CUVAF and myopia remained consistent within the older age stratum (Supplementary Table 2).

**Table 3:** Cross-tabulation of participants with high CUVAF (i.e., CUVAF area over the expected area for age) and myopia.

CUVAF category and diagnostic parameters	Myopia		
	Yes	No	
CUVAF area below the expected area for age	75 (TP)	620 (FP)	695
CUVAF area over the expected area for age	23 (FN)	261 (TN)	284
	98	881	979
Sensitivity = 75/98 = 0.765 (76.5%)			
Specificity = 261/881 = 0.296 (29.6%)			
Positive Predicted Value = 75/695 = 0.108 (10.79%)			
Negative Predicted Value = 261/284 = 0.919 (91.9%)			

Only subjects who responded to all these variables could be analyzed (979 of 1,129). The expected area for age is calculated using the previously published formula developed in the paper of de la Puente et al. (37).

Moreover, we evaluated the risk of developing either myopia or presenting with CUVAF depending on the time spent on OAs as continuous (per additional hour) and dichotomous (<7 vs. ≥7 h/week) variables (Table 4). For myopia, the odds ratio (OR) was 0.98 per additional hour of OAs (*p* = 0.01), indicating a 2% reduction in risk for each extra hour spent outdoors. Additionally, children engaging in ≥7 h of OAs per week had an OR of 0.50 (*p* < 0.001), corresponding to a 50% lower risk of developing myopia compared to those with less exposure.

**Table 4:** Odds ratio and 95% confidence interval of the association between time spent in outdoor activities and high CUVAF (i.e., CUVAF area over the expected area for age).

Exposure variables		OR	95% CI	*p* value
MYOPIA				
2° course	Per each additional hour spent in outdoor activities	0.99	0.97–1.01	0.652
	≥7 h/week vs. < 7 h/week in outdoor activities	1.13	0.38–3.38	>0.82
6° course	Per each additional hour spent in outdoor activities	0.98	0.969–0.994	0.005
	≥7 h/week vs. < 7 h/week in outdoor activities	0.41	0.27–0.62	<0.001
CUVAF				
2° course	Per each additional hour spent in outdoor activities	1.00	0.99–1.03	0.579
	≥7 h/week vs. < 7 h/week in outdoor activities	1.81	0.64–5.12	<0.001
6° course	Per each additional hour spent in outdoor activities	1.03	1.01–1.04	0.264
	≥7 h/week vs. < 7 h/week in outdoor activities	2.99	1.68–5.32	<0.001

Models were adjusted for age, sex, district, type of school (public vs. state-subsidized private), family history of myopia (yes vs. no), and electronic device use (<7 h/week vs. >7 h/week).

In contrast, for CUVAF, the OR was 1.02 per additional hour of OAs and 2.54 for ≥7 h/week, indicating that the likelihood of having CUVAF increases by 2% per hour of OA, and children with ≥7 h/week of OA had 2.54 times the odds (a 154% relative increase in odds) of having high CUVAF. Furthermore, the multivariate analysis of the relationship between the CUVAF biomarker and myopia demonstrated statistically significant findings (Table 5).

**Table 5:** Multivariate regression of myopia in patients with CUVAF+ compared to those with CUVAF.

Myopia	Odds ratio	St. error	p- value	95% IC (lower)	95% IC (upper)
CUVAF +	0.473	0.138	0.011	0.266	0.840

## 4 Discussion

The balance between NVAs and OAs has a decisive impact on the development and progression of myopia. These values provide clinicians with clear and actionable guidance for counseling families about myopia prevention.

The meta-analysis from Sherwin et al. demonstrated the protective effect of sunlight exposure, reporting a 2% risk reduction per additional hour spent outdoors, similar to our study, while the Raine cohort revealed an association between greater ocular sun exposure and lower myopia prevalence (17, 29, 39). Our results reinforce the growing consensus that increasing OAs is the most practical and easily modifiable strategy for reducing childhood myopia prevalence. Indeed, the observation that participants with greater outdoor activity also show a higher proportion of positive family history supports the idea that outdoor activity may act as a protective factor, even among individuals with a positive family history and, therefore, a higher genetic predisposition.

While outdoor activity is a well-established protective factor against myopia, prevention, their evaluation in clinical and research settings is heavily reliant on questionnaires, which are prone to recall bias and inconsistencies. CUVAF offers a transformative alternative, providing a rapid, objective, and non-invasive measure of cumulative UV exposure that can seamlessly be integrated into routine pediatric eye screenings. We have previously demonstrated a robust relationship between CUVAF, OAs, and myopia (8, 29, 37, 40), which is confirmed by our current findings.

For every additional hour of OAs, the probability of displaying an age-appropriate CUVAF area increases by 2%. More importantly, children with more than 7 h/week of OAs are 2.5 times more likely (a 144% probability) to reach or surpass the expected CUVAF threshold. These findings are consistent with the reported association between CUVAF and OAs and validate CUVAF as a reliable and objective biomarker for quantifying exposure to outdoor light.

CUVAF has a strong inverse correlation with myopia prevalence and severity. Children without myopia had significantly larger CUVAF areas than those with myopia, with the gradient being most pronounced in the moderate-to-high myopia subgroups (M2 and HM). These observations indicate that CUVAF not only reflects past UV exposure but may also identify children at risk of progressing to HM. In this study, an “adequate” CUVAF area confers a 91.9% probability of being non-myopic (negative predictive value). These predictive values are sufficiently robust for routine clinical use, and a simple CUVAF photograph could identify children who would benefit the most from targeted lifestyle counseling or early therapeutic intervention. It should be noted that the observed association between CUVAF and myopia does not imply that CUVAF itself is a protective factor. Rather, CUVAF serves as a biomarker of UV light exposure and, consequently, of outdoor activity, which are the parameters that truly act as protective factors against myopia. Moreover, when assessing the presence of CUVAF as a biomarker, it should be noted that its greatest utility lies in screening patients at low risk of developing myopia, but it would not be sufficient to establish a diagnosis of the condition.

Although this study provides valuable insights into the potential role of CUVAF as a biomarker for myopia, several limitations should be considered. The cross-sectional design precludes causal inference; longitudinal studies are warranted to determine whether increases in OAs (and CUVAF) actively slow axial elongation. We acknowledge that the use of a non-universal cycloplegia protocol might raise concerns regarding a potential risk of refractive error misclassification; however, any resulting bias is unlikely to meaningfully affect the prevalence estimates, as cycloplegia reduces the risk of misclassification by inhibiting accommodation; therefore, we consider cycloplegia sufficient when performed in patients with a refractive error below 0 before classifying them as myopic. The OA duration was self-reported, making it vulnerable to misclassification; however, an objective CUVAF measure provides indirect validation. Potential confounders, including diet, sunscreen use, and digital-screen time, were not fully accounted for, and categorizing continuous variables may have attenuated some correlations (41–43). Regarding sun-protection measures, their use was rare and did not affect CUVAF–outdoor associations. Nevertheless, the high sensitivity (76.5%) and negative predictive value (91.9%) of the age-adjusted CUVAF model support its use as a screening tool to identify children at risk of myopia. However, the low specificity (29.6%) suggests a high rate of false positives, underscoring the need for complementary diagnostic methods to confirm myopia risk in CUVAF-negative cases and ensure cost-effective, targeted interventions. This observation may be particularly pronounced in the present cohort, given that half of the participants are under 10 years of age and thus exhibit limited accumulation of CUVAF area. As reported in our previous studies (37), from the age of 12 years onward, the effectiveness of CUVAF is likely to be much higher in terms of both sensitivity and specificity, thereby improving the cost-effectiveness of CUVAF as a biomarker, which may be of interest for future research. Furthermore, it could be used to assess the degree of influence of environmental factors relative to genetics, as well as the potential prediction of the response to different myopia-control treatments.

To our knowledge, this is the first study to validate an age-specific CUVAF reference curve in a large and socioeconomically diverse pediatric population. CUVAF imaging is rapid, non-invasive, inexpensive, and highly acceptable to children. Additionally, its established association with OA and myopia risk positions it as a practical tool for early identification of at-risk individuals and for monitoring adherence to outdoor-time recommendations. Integrating CUVAF into school screening programs or routine ophthalmic examinations could enable data-driven, personalized strategies to reduce the burden of myopia on public health systems.

## 5 Conclusion

Our study confirms CUVAF as a reliable and objective biomarker of OA exposure and its inverse association with myopia severity in the pediatric population. In addition to its representativeness, the larger sample size of this study supports the potential clinical utility of CUVAF not only for evaluating an individual’s risk of myopia development during childhood but also for monitoring adherence to lifestyle modifications aimed at its prevention. The results also underscore the importance of incorporating objective measures, such as CUVAF, into early myopia management strategies and support their integration into clinical practice for personalized preventive approaches.

## Data Availability

The raw data supporting the conclusions of this article will be made available by the authors, without undue reservation.
