# Supplementary material for: More sun, less myopia: validating CUVAF as a biomarker for outdoor exposure in a cohort of Spanish schoolchildren
Source: Front Med (Lausanne). 2026 Feb 4;13:1708836. doi: 10.3389/fmed.2026.1708836 (PMC12913442; doi:10.3389/fmed.2026.1708836)
Supplement: Supplementary file 1 [file Data_Sheet_1.docx]

**Supplementary material**

**Supplementary Table 1.** Sociodemographic characteristics of full sample by myopia grade. OAH: Outdoor activity hours. IAH: Indoor activity hours.

| **Variable** | **Control (n=2164)** | **M1 (n=259)** | **M2 (n=53)** | **HM (n=13)** | **Total (n=2616)** |
| --- | --- | --- | --- | --- | --- |
| **Female (%)** | 1124 (52%) | 145 (44%) * | 18 (34%) * | 7 (54%) | **1331 (51%)** |
| **SE (D ± SD)** | 0.10 ± 0.23 | -1.20 ± 0.76 **** | -3.78 ± 0.60 **** | -7.4 ± 3.0 **** | **-0.24 ± 0.89** |
| **2nd Grade**  **(7 y/o) (%)** | 1110 (90%) | 65 (5.2%) | 4 (0.3%) | 4 (0.3%) | **1234 (100%)** |
| **6th Grade**  **(11 y/o) (%)** | 1054 (76%) *** | 194 (14%) *** | 49 (3.5%) *** | 9 (0.7%) | **1382 (100%)** |
| **CUVAF (%)** | 578 (88%) | 54 (8%) ** | 10 (1.5%) *** | 0 (0%) **** | **654 (100%)** |
| **OAH/w (± SD)** | 3.24 ± 2.38 | 2.91 ± 2.28 * | 2.25 ± 2.37 * | 2.07 ± 1.90 * | **3.05 ± 2.20** |
| **IAH/w (± SD)** | 17.2 ± 10.8 | 17.9 ± 10.6 | 16.6 ± 13.3 | 20.0 ± 9.1 | **17.2 ± 10.7** |

**Supplementary table 2**. Cross tabulation of participants with high CUVAF (i.e., CUVAF area over the expected area for age) and myopia 6th-grade subgroup. Only subjects who responded to all these variables could be analyzed (979 of the 1,129). The expected area for age is calculated using the previously published formula developed in the paper of de la Puente et al. (37)


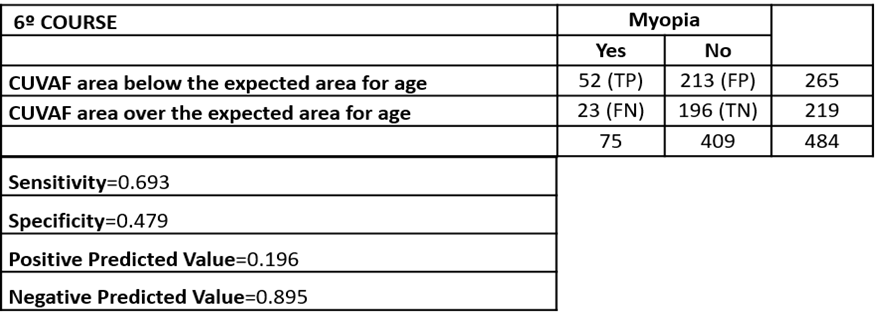


**Supplementary table 3.** Results of the area under the curve (AUROC) with 95% confidence intervals.

| **Variable** | **Coefficient** | **Std. Error** | **95% IC** |
| --- | --- | --- | --- |
| **AUC** | 0.547 | 0.033 | 0.484 – 0.611 |


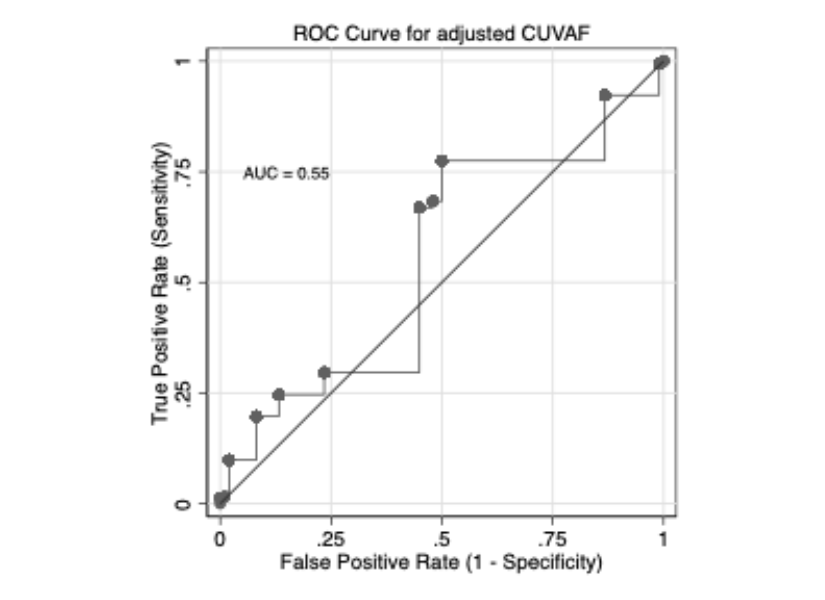
**Supplementary figure 1**. Area under the ROC curve (AUROC).
